# Defining the buffering process by a triprotic acid without relying on stewart-electroneutrality considerations

**DOI:** 10.1186/1742-4682-8-29

**Published:** 2011-08-17

**Authors:** Minhtri K Nguyen, Liyo Kao, Ira Kurtz

**Affiliations:** 1 Division of Nephrology, UCLA, Los Angeles, CA, USA

**Keywords:** acid, base, proton, Stewart

## Abstract

Upon the addition of protons to an aqueous solution, a component of the H^+ ^load will be bound i.e. buffered. In an aqueous solution containing a triprotic acid, H^+ ^can be bound to three different states of the acid as well as to OH^- ^ions that are derived from the auto-ionization of H_2_O. In quantifying the buffering process of a triprotic acid, one must define the partitioning of H^+ ^among the three states of the acid and also the OH^- ^ions in solution in order to predict the equilibrium pH value. However, previous quantitative approaches that model triprotic acid titration behaviour and used to predict the equilibrium pH rely on the mathematical convenience of electroneutrality/charge balance considerations. This fact has caused confusion in the literature, and has led to the assumption that charge balance/electroneutrality is a causal factor in modulating proton buffering (Stewart formulation). However, as we have previously shown, although charge balance can be used mathematically as a convenient tool in deriving various formulae, electroneutrality per se is not a fundamental physicochemical parameter that is mechanistically involved in the underlying buffering and proton transfer reactions. The lack of distinction between a mathematical tool, and a fundamental physicochemical parameter is in part a reason for the current debate regarding the Stewart formulation of acid-base analysis. We therefore posed the following question: Is it possible to generate an equation that defines and predicts the buffering of a triprotic acid that is based only on H^+ ^partitioning without incorporating electroneutrality in the derivation? Towards this goal, we derived our new equation utilizing: 1) partitioning of H^+ ^buffering; 2) conservation of mass; and 3) acid-base equilibria. In validating this model, we compared the predicted equilibrium pH with the measured pH of an aqueous solution consisting of Na_2_HPO_4 _to which HCl was added. The measured pH values were in excellent agreement with the predictions of our equation. Our results provide further important evidence that one can mathematically model the chemistry of acid-base phenomenology without relying on electroneutrality (Stewart formulation) considerations.

## 

Previous quantitative approaches have been derived that can accurately model the buffering process of a triprotic acid [1,2]. For example, electroneutrality requirement is a common mathematical tool that can be utilized to calculate the equilibrium pH of a triprotic acid-containing solution [2]. The n-bar equation is another mathematical tool used in modeling the buffering process of a triprotic acid which is derived based on the total bound proton fraction [1]. However, these previous mathematical approaches do not *mechanistically *define the *partitioning *of excess H^+ ^among the three different states of the acid as well as to OH^- ^ions that are derived from the auto-ionization of H_2_O. In this article, we present a new mathematical model that can mechanistically define the buffering process of a triprotic acid based on proton partitioning. We validate the model by comparing the predicted pH with the measured pH of a Na_2_HPO_4_-containing aqueous solution being titrated by HCl.

### Defining Proton Buffering by Triprotic Acids: Triprotic Acid Buffering Equation

$$H^{+}+A^{-3}\underset{}{⇆}HA^{-2}$$

Let *a *= concentration of excess *H*^+ ^buffered by *A*^-**3**^

$$H^{+}+HA^{-2}\underset{}{⇆}H_{2}A^{-1}$$

Let *b *= concentration of excess *H*^+ ^buffered by *HA*^-**2**^

$$H^{+}+H_{2}A^{-1}\underset{}{⇆}H_{3}A$$

Let *c *= concentration of excess *H*^+ ^buffered by *H*_**2**_*A*^-**1**^

$$H^{+}+OH^{-}\underset{}{⇆}H_{2}O$$

Let *d *= concentration of excess *H*^+ ^buffered by *OH*^-^

(1) $${\left[A^{-3}\right]}_{e}={\left[A^{-3}\right]}_{i}-a$$

(1a) $$∴a={\left[A^{-3}\right]}_{i}-{\left[A^{-3}\right]}_{e}$$

(2) $$\begin{array}{c} {\left[HA^{-2}\right]}_{e}={\left[HA^{-2}\right]}_{i}+a-b\\ ={\left[HA^{-2}\right]}_{i}+{\left[A^{-3}\right]}_{i}-{\left[A^{-3}\right]}_{e}-b \end{array}$$

(2a) $$∴b={\left[HA^{-2}\right]}_{i}-{\left[HA^{-2}\right]}_{e}+{\left[A^{-3}\right]}_{i}-{\left[A^{-3}\right]}_{e}$$

(3) $${\left[H_{3}A\right]}_{e}={\left[H_{3}A\right]}_{i}+c$$

(3a) $$∴c={\left[H_{3}A\right]}_{e}-{\left[H_{3}A\right]}_{i}$$

(4) $${\left[OH^{-}\right]}_{e}={\left[OH^{-}\right]}_{i}-d$$

(4a) $$∴d={\left[OH^{-}\right]}_{i}-{\left[OH^{-}\right]}_{e}={\left[OH^{-}\right]}_{i}-\frac{K^{\prime }w}{{\left[H^{+}\right]}_{e}}$$

where the suffix "*e*" stands for "equilibrium", suffix "*i*" stands for "initial", and *K'w *represents the dissociation constant of H_2_O.

Equilibrium [*H*^+^] must be equal to the difference between the initial [*H*^+^] and the sum of the concentrations of *H*^+ ^buffered by each proton acceptor site:

(5) $${\left[H^{+}\right]}_{e}={\left[H^{+}\right]}_{i}-a-b-c-d$$

$${\left[H^{+}\right]}_{e}={\left[H^{+}\right]}_{i}+{\left[A^{-3}\right]}_{e}-{\left[A^{-3}\right]}_{i}+{\left[HA^{-2}\right]}_{e}$$

(5a) $$\begin{array}{c} -{\left[HA^{-2}\right]}_{i}-{\left[A^{-3}\right]}_{i}+{\left[A^{-3}\right]}_{e}+{\left[H_{3}A\right]}_{i}-{\left[H_{3}A\right]}_{e}\\ +{\left[OH^{-}\right]}_{e}-{\left[OH^{-}\right]}_{i} \end{array}$$

$${\left[H^{+}\right]}_{e}={\left[H^{+}\right]}_{i}+\text{2}{\left[A^{-3}\right]}_{e}-\text{2}{\left[A^{-3}\right]}_{i}+{\left[HA^{-2}\right]}_{e}$$

(5b) $$-{\left[HA^{-2}\right]}_{i}+{\left[H_{3}A\right]}_{i}-{\left[H_{3}A\right]}_{e}+\frac{K^{\prime }w}{{\left[H^{+}\right]}_{e}}-\frac{K^{\prime }w}{{\left[H^{+}\right]}_{i}}$$

We will now solve for [*A*^-**3**^]_e_, [*HA*^-**2**^]_e_, and [*H*_**3**_*A*]_e_.

(6) $$A_{tot}={\left[A^{-3}\right]}_{e}+{\left[HA^{-2}\right]}_{e}+{\left[H_{2}A^{-1}\right]}_{e}+{\left[H_{3}A\right]}_{e}$$

(7) $${\left[H_{2}A^{-1}\right]}_{e}=\frac{K_{1}^{\prime }{\left[H_{3}A\right]}_{e}}{{\left[H^{+}\right]}_{e}}$$

(8) $${\left[HA^{-2}\right]}_{e}=\frac{K_{2}^{\prime }{\left[H_{2}A^{-1}\right]}_{e}}{{\left[H^{+}\right]}_{e}}=\frac{K_{1}^{\prime }K_{2}^{\prime }{\left[H_{3}A\right]}_{e}}{{\left[H^{+}\right]}_{e}^{2}}$$

(9) $${\left[A^{-3}\right]}_{e}=\frac{K_{3}^{\prime }{\left[HA^{-2}\right]}_{e}}{{\left[H^{+}\right]}_{e}}=\frac{K_{1}^{\prime }K_{2}^{\prime }K_{3}^{\prime }{\left[H_{3}A\right]}_{e}}{{\left[H^{+}\right]}_{e}^{3}}$$

(6a) $$∴\left[A_{tot}\right]=\frac{K_{1}^{\prime }K_{2}^{\prime }K_{3}^{\prime }{\left[H_{3}A\right]}_{e}}{{\left[H^{+}\right]}_{e}^{3}}+\frac{K_{1}^{\prime }K_{2}^{\prime }{\left[H_{3}A\right]}_{e}}{{\left[H^{+}\right]}_{e}^{2}}+\frac{K_{1}^{\prime }{\left[H_{3}A\right]}_{e}}{{\left[H^{+}\right]}_{e}}+{\left[H_{3}A\right]}_{e}$$

where *K'_1_, K'_2_*, and *K'_3 _*represent the three dissociation constants of the triprotic acid.

Solving for [*H*_**3**_*A*]_e_:

(10) $${\left[H_{3}A\right]}_{e}=\frac{{\left[H^{+}\right]}_{e}^{3}\left(\left[A_{tot}\right]\right)}{K_{1}^{\prime }K_{2}^{\prime }K_{3}^{\prime }+K_{1}^{\prime }K_{2}^{\prime }{\left[H^{+}\right]}_{e}+K_{1}^{\prime }{\left[H^{+}\right]}_{e}^{2}+{\left[H^{+}\right]}_{e}^{3}}$$

Entering Eq.10 into Eq.9:

(9a) $${\left[A^{-3}\right]}_{e}=\frac{\left(K_{1}^{\prime }K_{2}^{\prime }K_{3}^{\prime }\right)\left(\left[A_{tot}\right]\right)}{K_{1}^{\prime }K_{2}^{\prime }K_{3}^{\prime }+K_{1}^{\prime }K_{2}^{\prime }{\left[H^{+}\right]}_{e}+K_{1}^{\prime }{\left[H^{+}\right]}_{e}^{2}+{\left[H^{+}\right]}_{e}^{3}}$$

Entering Eq.10 into Eq.8:

(8a) $${\left[HA^{-2}\right]}_{e}=\frac{\left(K_{1}^{\prime }K_{2}^{\prime }\right)\left({\left[H^{+}\right]}_{e}\left[A_{tot}\right]\right)}{K_{1}^{\prime }K_{2}^{\prime }K_{3}^{\prime }+K_{1}^{\prime }K_{2}^{\prime }{\left[H^{+}\right]}_{e}+K_{1}^{\prime }{\left[H^{+}\right]}_{e}^{2}+{\left[H^{+}\right]}_{e}^{3}}$$

Entering Eq.8a, 9a, and Eq.10 into Eq.5b:

(5c) $$\begin{array}{c} {\left[H^{+}\right]}_{e}={\left[H^{+}\right]}_{i}+\frac{\left(\text{2}K_{1}^{\prime }K_{2}^{\prime }K_{3}^{\prime }\right)\left(\left[A_{tot}\right]\right)}{K_{1}^{\prime }K_{2}^{\prime }K_{3}^{\prime }+K_{1}^{\prime }K_{2}^{\prime }{\left[H^{+}\right]}_{e}+K_{1}^{\prime }{\left[H^{+}\right]}_{e}^{2}+{\left[H^{+}\right]}_{e}^{3}}\\ -\text{2}{\left[A^{-3}\right]}_{i}+\frac{\left(K_{1}^{\prime }K_{2}^{\prime }\right)\left({\left[H^{+}\right]}_{e}\left[A_{tot}\right]\right)}{K_{1}^{\prime }K_{2}^{\prime }K_{3}^{\prime }+K_{1}^{\prime }K_{2}^{\prime }{\left[H^{+}\right]}_{e}+K_{1}^{\prime }{\left[H^{+}\right]}_{e}^{2}+{\left[H^{+}\right]}_{e}^{3}}\\ -{\left[HA^{-2}\right]}_{i}+{\left[H_{3}A\right]}_{i}-\frac{{\left[H^{+}\right]}_{e}^{3}\left(\left[A_{tot}\right]\right)}{K_{1}^{\prime }K_{2}^{\prime }K_{3}^{\prime }+K_{1}^{\prime }K_{2}^{\prime }{\left[H^{+}\right]}_{e}+K_{1}^{\prime }{\left[H^{+}\right]}_{e}^{2}+{\left[H^{+}\right]}_{e}^{3}}+\frac{K^{\prime }w}{{\left[H^{+}\right]}_{e}}-\frac{K^{\prime }w}{{\left[H^{+}\right]}_{i}} \end{array}$$

Solving Eq.5c for [*H*^+^]_e_:

(5d) $$\begin{array}{l} {\left[H^{+}\right]}_{e}^{5}\\ +{\left[H^{+}\right]}_{e}^{4}(K_{1}^{\prime }-{\left[H^{+}\right]}_{i}+\text{2}{\left[A^{-3}\right]}_{i}+{\left[HA^{-2}\right]}_{i}-{\left[H_{3}A\right]}_{i}\\ +[A_{tot}]+\frac{K^{\prime }w}{{\left[H^{+}\right]}_{i}})\\ +{\left[H^{+}\right]}_{e}^{3}(K_{1}^{\prime }K_{2}^{\prime }-K_{1}^{\prime }{\left[H^{+}\right]}_{i}+\text{2}K_{1}^{\prime }{\left[A^{-3}\right]}_{i}\\ +K_{1}^{\prime }{\left[HA^{-2}\right]}_{i}-K_{1}^{\prime }{\left[H_{3}A\right]}_{i}-K^{\prime }w+\frac{K_{1}^{\prime }K^{\prime }w}{{\left[H^{+}\right]}_{i}})\\ +{\left[H^{+}\right]}_{e}^{2}(K_{1}^{\prime }K_{2}^{\prime }K_{3}^{\prime }-K_{1}^{\prime }K_{2}^{\prime }{\left[H^{+}\right]}_{i}+\text{2}K_{1}^{\prime }K_{2}^{\prime }{\left[A^{-3}\right]}_{i}\\ -K_{1}^{\prime }K_{2}^{\prime }[A_{tot}]+K_{1}^{\prime }K_{2}^{\prime }{\left[HA^{-2}\right]}_{i}-K_{1}^{\prime }K_{2}^{\prime }{\left[H_{3}A\right]}_{i}\\ -K_{1}^{\prime }K^{\prime }w+\frac{K_{1}^{\prime }K_{2}^{\prime }K^{\prime }w}{{\left[H^{+}\right]}_{i}})\\ +{\left[H^{+}\right]}_{e}(\text{2}K_{1}^{\prime }K_{2}^{\prime }K_{3}^{\prime }{\left[A^{-3}\right]}_{i}-K_{1}^{\prime }K_{2}^{\prime }K_{3}^{\prime }{\left[H^{+}\right]}_{i}\\ -\text{2}K_{1}^{\prime }K_{2}^{\prime }K_{3}^{\prime }[A_{tot}]+K_{1}^{\prime }K_{2}^{\prime }K_{3}^{\prime }{\left[HA^{-2}\right]}_{i}\\ -K_{1}^{\prime }K_{2}^{\prime }K_{3}^{\prime }{\left[H_{3}A\right]}_{i}-K_{1}^{\prime }K_{2}^{\prime }K^{\prime }w+\frac{K_{1}^{\prime }K_{2}^{\prime }K_{3}^{\prime }K^{\prime }w}{{\left[H^{+}\right]}_{i}})\\ -K_{1}^{\prime }K_{2}^{\prime }K_{3}^{\prime }K^{\prime }w=\text{0} \end{array}$$

Solve Eq. 5d for "[H^+^]_e_" by finding the roots of a fifth-order polynomial equation using the wxMaxima software.

The apparent equilibrium constant *K' *is calculated based on the thermodynamic equilibrium constant *K *according to the following equation [3,4]:

(11) $$pK^{\prime }=pK-0.51\sqrt{I}$$

where *I *= ionic strength

(12) I=12∑cizi2

where c_i _is the molar concentration of each ion and z_i _is its charge.

## Methods

In validating the Triprotic Acid Buffering Equation (TABE), we tested the formula using a Na_2_HPO_4_-containing aqueous solution (Sigma, St. Louis, MO) and measured the pH changes following the addition of HCl. To obtain pH measurements, the pH electrode (Sensorex, Garden Grove, CA) was calibrated using standard buffers of pH 1.68 (Ricca Chemical Company, Arlington, TX), pH of 4.01 and 7.00 (Thermo Electron Corporation, Beverly, MA). 20 ml of a 10 mM Na_2_HPO_4_-containing aqueous solution was incubated at 25°C in a water bath (Thermo Fisher Scientific, Waltham, MA). Then, 20 μl of 1.0 M HCl (Sigma, St. Louis, MO) was added to the solution, and the equilibrium pH was measured when there was no further change in the measured pH with time. To obtain more equilibrium pH values, the addition of 20 μl of 1.0 M HCl was repeated. The pH meter (Hanna Instruments, Woonsocket, RI) was calibrated at 25°C, and the equilibrium pH was measured utilizing a pH electrode at 25°C while the solution was mixed.

At each titration step, the reactant concentrations of each sample were first calculated based on the *measured *pH of the sample *prior *to the addition of HCl according to Equations 8a, 9a and 10.

Based on the water association/dissociation equilibrium reaction:

$${\left[\text{O}{\text{H}}^{-}\right]}_{\text{sample}}=K_{w}^{\prime }∕{\left[{\text{H}}^{+}\right]}_{\text{sample}}$$

*After *the addition of HCl, the *initial *reactant concentrations as displayed in Table 1 were calculated by accounting for the amount of H^+ ^and OH^- ^added and the dilutional effect of the added volume:

**Table 1:** Predicted pH vs. Measured pH

[H^+^]_i _mol/L	[OH^-^]_i _mol/L	K'w	K'1	K'2	K'3	[H_3_A]_i _mol/L	[HA^-2^]_i _mol/L	[A^-3^]_i _mol/L	A_tot _mol/L	Predicted [H^+^]_e_	Predicted pH_e_	Measured pH_e_
9.9900E-04	1.2870E-05	1.2206E-14	9.2591E-03	7.5261E-08	5.8421E-13	1.2702E-11	9.8598E-03	6.0795E-06	9.9900E-03	9.3446E-09	8.03	7.92
9.9802E-04	8.3093E-07	1.2128E-14	9.2003E-03	7.4783E-08	5.8050E-13	1.7940E-09	8.6018E-03	4.1684E-07	9.9800E-03	2.3305E-08	7.63	7.57
9.9704E-04	3.6692E-07	1.2055E-14	9.1447E-03	7.4331E-08	5.7699E-13	7.9068E-09	7.3044E-03	1.5537E-07	9.9701E-03	4.3085E-08	7.37	7.32
9.9606E-04	2.0872E-07	1.1988E-14	9.0941E-03	7.3920E-08	5.7380E-13	2.0475E-08	6.0547E-03	7.2865E-08	9.9602E-03	7.1549E-08	7.15	7.12
9.9510E-04	1.3056E-07	1.1930E-14	9.0498E-03	7.3559E-08	5.7100E-13	4.2642E-08	4.8869E-03	3.6612E-08	9.9502E-03	1.1446E-07	6.94	6.92
9.9416E-04	8.3333E-08	1.1877E-14	9.0099E-03	7.3236E-08	5.6849E-13	8.1648E-08	3.7791E-03	1.7993E-08	9.9404E-03	1.8817E-07	6.73	6.72
9.9324E-04	5.1978E-08	1.1831E-14	8.9747E-03	7.2949E-08	5.6627E-13	1.5335E-07	2.7402E-03	8.1067E-09	9.9305E-03	3.4187E-07	6.47	6.48
9.9239E-04	3.0170E-08	1.1792E-14	8.9450E-03	7.2708E-08	5.6439E-13	2.9964E-07	1.7922E-03	3.0675E-09	9.9206E-03	8.2868E-07	6.08	6.15
9.9168E-04	1.6510E-08	1.1773E-14	8.9309E-03	7.2593E-08	5.6350E-13	5.9702E-07	1.0652E-03	9.9569E-10	9.9108E-03	7.9634E-06	5.10	5.32
9.9487E-04	2.0932E-09	1.1795E-14	8.9472E-03	7.2725E-08	5.6453E-13	5.2047E-06	1.4838E-04	1.7534E-11	9.9010E-03	4.1477E-04	3.38	3.37
1.4202E-03	2.3151E-11	1.1817E-14	8.9638E-03	7.2860E-08	5.6558E-13	4.5503E-04	1.5900E-06	2.0801E-15	9.8912E-03	9.3769E-04	3.03	3.00
1.9900E-03	9.9614E-12	1.1840E-14	8.9816E-03	7.3005E-08	5.6670E-13	9.9425E-04	6.4561E-07	3.6409E-16	9.8814E-03	1.5388E-03	2.81	2.79
2.5934E-03	6.2133E-12	1.1865E-14	9.0006E-03	7.3160E-08	5.6790E-13	1.4988E-03	3.8016E-07	1.3399E-16	9.8717E-03	2.1727E-03	2.66	2.65
3.2163E-03	4.4753E-12	1.1892E-14	9.0208E-03	7.3324E-08	5.6917E-13	1.9597E-03	2.5898E-07	6.5889E-17	9.8619E-03	2.8245E-03	2.55	2.54
3.8914E-03	3.4342E-12	1.1920E-14	9.0421E-03	7.3497E-08	5.7052E-13	2.4023E-03	1.8779E-07	3.6744E-17	9.8522E-03	3.5284E-03	2.45	2.45
4.5699E-03	2.7834E-12	1.1949E-14	9.0641E-03	7.3676E-08	5.7191E-13	2.7966E-03	1.4429E-07	2.2936E-17	9.8425E-03	4.2333E-03	2.37	2.37
5.2942E-03	2.3151E-12	1.1979E-14	9.0871E-03	7.3862E-08	5.7335E-13	3.1711E-03	1.1375E-07	1.5077E-17	9.8328E-03	4.9831E-03	2.30	2.30
5.9749E-03	1.9991E-12	1.2009E-14	9.1100E-03	7.4049E-08	5.7480E-13	3.4854E-03	9.3674E-08	1.0747E-17	9.8232E-03	5.6856E-03	2.25	2.24
6.7136E-03	1.7411E-12	1.2041E-14	9.1338E-03	7.4242E-08	5.7631E-13	3.7922E-03	7.7712E-08	7.7855E-18	9.8135E-03	6.4459E-03	2.19	2.18
7.5241E-03	1.5252E-12	1.2073E-14	9.1587E-03	7.4444E-08	5.7787E-13	4.0941E-03	6.4726E-08	5.6956E-18	9.8039E-03	7.2775E-03	2.14	2.13
8.3216E-03	1.3593E-12	1.2106E-14	9.1835E-03	7.4647E-08	5.7944E-13	4.3602E-03	5.5049E-08	4.3289E-18	9.7943E-03	8.0938E-03	2.09	2.09
9.1457E-03	1.2220E-12	1.2149E-14	9.2161E-03	7.4911E-08	5.8149E-13	4.6079E-03	4.7274E-08	3.3510E-18	9.7847E-03	8.9351E-03	2.05	2.05
9.9070E-03	1.1177E-12	1.2172E-14	9.2334E-03	7.5052E-08	5.8259E-13	4.8147E-03	4.1542E-08	2.7005E-18	9.7752E-03	9.7110E-03	2.01	2.01
1.0711E-02	1.0253E-12	1.2205E-14	9.2585E-03	7.5256E-08	5.8417E-13	5.0141E-03	3.6598E-08	2.1882E-18	9.7656E-03	1.0529E-02	1.98	1.97
1.1588E-02	9.4044E-13	1.2239E-14	9.2844E-03	7.5467E-08	5.8581E-13	5.2125E-03	3.2191E-08	1.7705E-18	9.7561E-03	1.1420E-02	1.94	1.94

$$\left[A_{tot}\right]=\frac{\left(\text{0}\text{.01}\times \text{0}\text{.02}\right)}{TotalVol}$$

where *Vol *= volume

$${\left[A^{-3}\right]}_{i}=\frac{\left({\left[A^{-3}\right]}_{sample}\times Vol_{sample}\right)}{TotalVol}$$

$${\left[HA^{-2}\right]}_{i}=\frac{\left({\left[HA^{-2}\right]}_{sample}\times Vol_{sample}\right)}{TotalVol}$$

$${\left[H_{3}A\right]}_{i}=\frac{\left({\left[H_{3}A\right]}_{sample}\times Vol_{sample}\right)}{TotalVol}$$

$${\left[H^{+}\right]}_{i}=\frac{\left({\left[H^{+}\right]}_{sample}\times Vol_{sample}+{\left[H^{+}\right]}_{HCl}\times Vol_{HCl}\right)}{TotalVol}$$

where [*H*^+^]*_HCl_*= *H*^+ ^concentration of HCl solution; *Vol_HCl_*= volume of HCl added; and HCl is assumed to be completely dissociated.

$${\left[OH^{-}\right]}_{i}=\frac{\left({\left[OH^{-}\right]}_{sample}\times Vol_{sample}+{\left[OH^{-}\right]}_{HCl}\times Vol_{HCl}\right)}{TotalVol}$$

where [*OH*^-^]*_HCl _= OH*^- ^concentration of the HCl solution $=\frac{K^{\prime }w}{{\left[H^{+}\right]}_{HCl}}$

## Results

### Comparison of predicted pH with measured pH

In calculating the predicted equilibrium [H^+^] of a Na_2_HPO_4_-containing aqueous solution being titrated by HCl, the equilibrium pH is first estimated using the thermodynamic equilibrium constant *K *(expressed in terms of activities) of each of the three different states of the acid as well as that of H_2_O, and this pH is then used to estimate the ionic strength at equilibrium [5-7]. The final equilibrium pH is then re-calculated using the apparent equilibrium constant *K' *(expressed in terms of molar concentrations) which is calculated based on the predicted ionic strength at equilibrium according to Equation 11 [3]. The apparent equilibrium constant *K' *is utilized to predict the final equilibrium pH since the reactant concentrations are expressed in molar concentrations.

**Figure 1:**
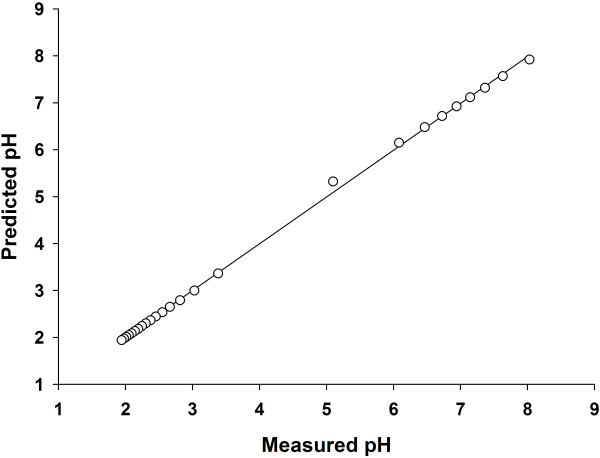
**Comparison of predicted equilibrium pH with the measured equilibrium pH**. The linear least squares fit equation comparing predicted to measured pH had an r^2 ^value of 1.00, slope of 1.003, and an intercept of-0.007.

In validating the Triprotic Acid Buffering Equation (TABE), we compared the predicted equilibrium pH as calculated by TABE with the measured pH (mean of eight measurements) of an aqueous solution consisting of Na_2_HPO_4 _to which HCl was added. As demonstrated in Figure 1 and Table 1, the predicted equilibrium pH as calculated by TABE accurately predicted the measured equilibrium pH values. The linear least squares fit equation comparing all predicted to measured values had an r^2 ^value of 1.0, with a slope of 1.003, and an intercept of-0.007 (Figure 1). Not only is the predicted pH as calculated by TABE accurate in predicting the equilibrium pH, we also show its utility in defining the partitioning of H^+ ^among the three different states of the acid as well as to OH^- ^ions that are derived from the auto-ionization of H_2_O (Table 2).

**Table 2:** Partitioning of H^+ ^Buffering

[H^+^]_i _mol/L	H^+ ^buffered by A^-3 ^mol/L	H^+ ^buffered by HA^-2 ^mol/L	H^+ ^buffered by HA^-1 ^mol/L	H^+ ^buffered by OH^- ^mol/L	[H^+^]_e _mol/L
9.9900E-04	5.5256E-06	9.8190E-04	1.1066E-09	1.1567E-05	9.3446E-09
9.9802E-04	2.2785E-07	9.9745E-04	4.2367E-09	3.1171E-07	2.3305E-08
9.9704E-04	7.1046E-08	9.9683E-04	9.3780E-09	8.7597E-08	4.3085E-08
9.9606E-04	3.2334E-08	9.9590E-04	1.8123E-08	4.1327E-08	7.1549E-08
9.9510E-04	1.7204E-08	9.9491E-04	3.4008E-08	2.6372E-08	1.1446E-07
9.9416E-04	9.5770E-09	9.9387E-04	6.7756E-08	2.0201E-08	1.8817E-07
9.9324E-04	5.2097E-09	9.9272E-04	1.5808E-07	1.7342E-08	3.4187E-07
9.9239E-04	2.5210E-09	9.9100E-04	5.4393E-07	1.5920E-08	8.2868E-07
9.9168E-04	9.8934E-10	9.7557E-04	8.1389E-06	1.5029E-08	7.9634E-06
9.9487E-04	1.7532E-11	1.4672E-04	4.3338E-04	2.0648E-09	4.1477E-04
1.4202E-03	1.6605E-15	8.9429E-07	4.8163E-04	1.0549E-11	9.3769E-04
1.9900E-03	2.1669E-16	2.4538E-07	4.5100E-04	2.2669E-12	1.5388E-03
2.5934E-03	6.3997E-17	1.1239E-07	4.2066E-04	7.5201E-13	2.1727E-03
3.2163E-03	2.6596E-17	6.4006E-08	3.9169E-04	2.6485E-13	2.8245E-03
3.8914E-03	1.2872E-17	4.0158E-08	3.6298E-04	5.5708E-14	3.5284E-03
4.5699E-03	7.1587E-18	2.7510E-08	3.3659E-04	-3.9428E-14	4.2333E-03
5.2942E-03	4.2453E-18	1.9614E-08	3.1116E-04	-8.8908E-14	4.9831E-03
5.9749E-03	2.7824E-18	1.4894E-08	2.8926E-04	-1.1333E-13	5.6856E-03
6.7136E-03	1.8591E-18	1.1434E-08	2.6769E-04	-1.2711E-13	6.4459E-03
7.5241E-03	1.2572E-18	8.8354E-09	2.4657E-04	-1.3397E-13	7.2775E-03
8.3216E-03	8.9060E-19	7.0279E-09	2.2781E-04	-1.3658E-13	8.0938E-03
9.1457E-03	6.4555E-19	5.6698E-09	2.1061E-04	-1.3664E-13	8.9351E-03
9.9070E-03	4.9118E-19	4.7172E-09	1.9601E-04	-1.3578E-13	9.7110E-03
1.0711E-02	3.7582E-19	3.9347E-09	1.8196E-04	-1.3404E-13	1.0529E-02
1.1588E-02	2.8736E-19	3.2799E-09	1.6860E-04	-1.3132E-13	1.1420E-02

## Discussion

Significant changes in the [H^+^] resulting from the addition of a proton load to a triprotic acid-containing aqueous solution are prevented by the process of H^+ ^buffering. The equilibrium pH of a Na_2_HPO_4_-containing aqueous solution is therefore determined by the buffering of excess H^+ ^by the various phosphate acceptor sites as well as to OH^- ^ions that are derived from the auto-ionization of H_2_O. Historically, the Guenther's n-bar equation has been used to predict the equilibrium pH of a Na_2_HPO_4_-containing aqueous solution [1]. The Guenther's n-bar equation is based on the *total *bound proton fraction at equilibrium. Therefore, the Guenther's n-bar equation accounts for both *pre-existing *bound phosphate species as well as *newly *bound phosphate species that are formed from the buffering of excess H^+ ^by the various phosphate acceptor sites. As the Guenther's n-bar equation is based on the *total *bound proton fraction, it is simply a convenient mathematical tool for predicting equilibrium pH. Specifically, its derivation is not *mechanistically *based since it does not define the *partitioning *of proton buffering by determining only the *newly *bound phosphate species that are formed from the buffering of excess H^+^.

Similarly, electroneutrality based on charge balance requirement has also been utilized to define the equilibrium pH of a triprotic acid-containing aqueous solution [2]. However, we had previously shown that although *charge balance is a convenient mathematical tool *that can be utilized to calculate and predict the equilibrium pH, charge balance (electroneutrality considerations) is not a *fundamental physicochemical parameter *that is *mechanistically *involved in predicting or determining the equilibrium pH value of a solution [4,8,9] Indeed, if strong ion difference (SID, a term used in the Stewart formulation which is based on electroneutrality and charge balance considerations) were to have a *mechanistic *role in determining the equilibrium pH, it must do so by *imparting a fixed macroscopic charge *to the solution *which will in turn cause the [H^+^] to attain a given value in order to maintain macroscopic electroneutrality *[8,9]. However, we demonstrated that for a given change in SID due to the addition of HCl to a NaCl-containing solution, electroneutrality is maintained (i.e. [Na^+^] + [H^+^]-[Cl^-^]-[OH^-^] = 0) at all pre-equilibrium and equilibrium pH values, and that the equilibrium pH is only determined by the dissociation constant of water, *K'_w_*[8,9]. In this previous study, we also reported the derivation of a new mathematical model to predict the equilibrium pH based *mechanistically *on the partitioning of H^+ ^buffering in an aqueous solution containing multiple *monoprotic *acids without relying on electroneutrality requirements [8]. The goal of our present study is to determine whether it is possible to derive a mathematical model based on the underlying physical chemistry involved (i.e. partitioning of H^+ ^buffering) in a *triprotic acid*-containing aqueous solution without utilizing the mathematical convenience of electroneutrality/charge balance considerations as had previous authors. Our reasoning was based on the consideration that if a derivation based only on partitioning of H^+ ^buffering was indeed possible in predicting the equilibrium pH of a triprotic acid-containing aqueous solution as well, this would demonstrate convincingly that electroneutrality/charge balance considerations are not only mathematically not required, but are de facto not fundamental in determining the pH from a chemical standpoint.

Towards this goal, we derived our new mathematical model utilizing: 1) partitioning of H^+ ^buffering; 2) conservation of mass; and 3) acid-base equilibria. Simply stated, in an aqueous solution containing a triprotic acid H_3_A, the equilibrium [H^+^] must be equal to

the difference between the initial [H^+^] and the sum of the concentrations of H^+ ^buffered by each proton acceptor site:

$${\left[H^{+}\right]}_{e}={\left[H^{+}\right]}_{i}-a-b-c-d$$

where:

*a *= concentration of excess *H*^+ ^buffered by *A*^-**3**^

*b *= concentration of excess *H*^+ ^buffered by *HA*^-**2**^

*c *= concentration of excess *H*^+ ^buffered by *HA*^-**1**^

*d *= concentration of excess *H*^+ ^buffered by *OH*^-^

Our model is also based on the law of conservation of mass:

$$A_{tot}={\left[A^{-3}\right]}_{e}+{\left[HA^{-2}\right]}_{e}+{\left[H_{2}A^{-1}\right]}_{e}+{\left[H_{3}A\right]}_{e}$$

Based on the acid-base equilibrium reactions of Na_2_HPO_4 _and H_2_O and the above mathematical relationships, we derived a fifth-order polynomial equation which can be utilized to predict the equilibrium [H^+^] of a triprotic acid-containing aqueous solution. The five possible roots of this fifth-order polynomial equation can be easily computed using user-friendly mathematical software such as wxMaxima. Although there are five possible roots to this polynomial equation, there is only one solution that is positive in value.

### Validity of TABE in Predicting the Equilibrium pH

In validating our new mathematical model, we tested the model using a Na_2_HPO_4_-containing solution and measured the pH changes following the addition of HCl. In our study, HCl was added successively to a Na_2_HPO_4_-containing solution and the equilibrium pH is measured at each addition step. The equilibrium pH was determined when there was no further change in the measured pH with time at each step of HCl addition. The measured equilibrium pH was then compared with the predicted equilibrium pH as calculated by our model. In predicting the equilibrium pH, the equilibrium pH was first estimated using the thermodynamic equilibrium constant *K *(expressed in terms of activities) of each of the three different states of the acid as well as that of H_2_O, and this pH value was then used to estimate the ionic strength at equilibrium [5-7]. The final equilibrium pH was then re-calculated using the apparent equilibrium constant *K' *(expressed in terms of molar concentrations) which was calculated based on the predicted ionic strength at equilibrium according to Equation 11 [3]. The apparent equilibrium constant *K' *was utilized to calculate the final equilibrium pH since the reactant concentrations were expressed in molar concentrations. As demonstrated in Table 1 and Figure 1, our results confirmed the accuracy of our new quantitative approach for predicting the final equilibrium pH. Indeed, linear regression analysis demonstrated that the predicted pH as calculated by our model is in excellent agreement with the measured pH:

$$\text{Predicted pH}=1.00\text{3 Measured pH}-0.00\text{7 }\left({\text{r}}^{2}=1.0\right)$$

Our new mathematical model is also an important tool for defining the buffering of excess H^+ ^among the various proton acceptor sites in solution. As shown in Table 2, by defining the partitioning of H^+ ^buffering among the different proton acceptor sites, the equilibrium [H^+^] is simply determined from a chemical standpoint by the difference between the initial [H^+^] and the sum of the concentrations of H^+ ^being buffered by each of the individual proton acceptor sites.

Theoretically, our formula is also valid for defining the buffering of excess H^+ ^by other triprotic acids, eg. tricarboxylic acid such as citrate, an amino acid such as lysine, etc. Future studies can easily be performed to test the accuracy of our model in defining the buffering processes by other triprotic acids.

### Potential Sources of Error

In calculating the predicted equilibrium pH, the value of the thermodynamic equilibrium pK of each proton acceptor site was based on reported values at 25 degrees C [5,6]. In refining the predictive accuracy of our model, the apparent equilibrium *pK' *was utilized to calculate the final equilibrium pH since the reactant concentrations were expressed in molar concentrations [3]. The apparent equilibrium *pK' *was calculated based on the thermodynamic equilibrium pK and the predicted ionic strength of the solution at equilibrium according to Equation 11 [3]. Consequently, errors in either the reported values and/or the correction for ionic strength would introduce discrepancies between predicted and measured values. An additional source of error is in the accuracy of the pH electrode measurements. However, despite these potential sources of error, the measured values were not significantly different from the values predicted using our formula.

## Summary

Our new mathematical model is the first reported quantitative approach that can predict the equilibrium [H^+^] based *mechanistically *on the *partitioning *of H^+ ^buffering among the different proton acceptor sites of a triprotic acid-containing solution. As shown in Table 2, by defining the partitioning of H^+ ^buffering among the different proton acceptor sites, the equilibrium [H^+^] is *physicochemically *determined by the difference between the initial [H^+^] and the sum of the concentrations of H^+ ^being buffered by each of the individual proton acceptor sites. Importantly, our results demonstrate that the equilibrium pH can be accurately predicted based *mechanistically *on the *partitioning *of H^+ ^buffering among the proton acceptor sites in solution without requiring calculations or considerations based on electroneutrality requirements. These results therefore provide additional data demonstrating that electroneutrality requirements do not play any role in defining the equilibrium [H^+^] of a solution.

## Competing interests

The authors declare that they have no competing interests.

## Authors' contributions

MKN derived the mathematical model and drafted the manuscript. LK and IK conducted the buffer titration experiments. All authors read and approved the final version of the manuscript.
